# AN EXPLORATORY DESCRIPTIVE STUDY OF CARE PARTNERS’ PREFERENCES AND SKILL TRAINING NEEDS DURING HOSPITAL CARE

**DOI:** 10.1093/geroni/igac059.2354

**Published:** 2022-12-20

**Authors:** Kaitlyn Vanias, Andrea Yahr, Beth Fields

**Affiliations:** University of Wisconsin-Madison, Madison, Wisconsin, United States; Geriatric Health Services Research Lab, Madison, Wisconsin, United States; Geriatric Health Services Research Lab, Madison, Wisconsin, United States

## Abstract

Care partners play a critical role in caring for an increasingly complex, aging population in the United States after hospital discharge. However, care partners’ preferences and needs are often not formally assessed during their loved one’s hospitalization. The Care Partner Hospital Assessment Tool (CHAT) is a standardized decision-support tool that facilitates the inclusion and training of care partners during hospital care. The purpose of this exploratory descriptive study was to understand the preferences and needs of 12 care partners of older adult patients admitted to a large academic hospital utilizing the CHAT. The majority (8 of 12) of care partners surveyed were spouses while the remainder were adult children of the patients. All care partners provided comparable support including physical and social support as well as healthcare management. Despite providing similar support, there were differences in the care preferences and needs of spouses compared to adult children. A greater proportion of spouses preferred to be present for care in the hospital and requested information on adaptive equipment and community services. All adult children desired access to the electronic medical record while there was a mixed response among spouses. The most common needs across care partners included training on mobility assistance and medical devices. Findings from this study demonstrate that care partners have varying preferences and training needs during hospital care and suggest differences across care partner relationships. Further investigation is necessary to better understand these patterns and improve hospital care for patients and their care partners.

